# Differential Anti-Inflammatory Effects of Semaglutide and Tirzepatide in Experimental Diabetes Mellitus

**DOI:** 10.3390/cimb48070675

**Published:** 2026-06-30

**Authors:** Roxana-Cristina Dobriceanu, Ianis Kevyn Stefan Boboc, Liliana Mititelu Tartau, Andreea Daniela Meca, Carmen Nicoleta Oancea, Maria-Alexandra Paceana, Marius-Mihai Pastiu, Adina Turcu-Stiolica, Maria Bogdan

**Affiliations:** 1 Doctoral School, University of Medicine and Pharmacy, 200349 Craiova, Romania; dobriceanuroxana@yahoo.com (R.-C.D.); mariaalexandrapaceana@gmail.com (M.-A.P.); marius.pastiu95@gmail.com (M.-M.P.); 2 Department of Pharmacology, Faculty of Pharmacy, University of Medicine and Pharmacy, 200349 Craiova, Romania; andreea_mdc@yahoo.com (A.D.M.); bogdanfmaria81@yahoo.com (M.B.); 3 Grigore T. Popa University of Medicine and Pharmacy Iasi, 700115 Iasi, Romania; lylytartau@yahoo.com; 4 Department of Biochemistry, Faculty of Medicine, University of Medicine and Pharmacy, 200349 Craiova, Romania; carmen.oancea@umfcv.ro; 5 Department of Pharmaceutical Marketing and Management, Faculty of Pharmacy, University of Medicine and Pharmacy, 200349 Craiova, Romania; adina.turcu@gmail.com

**Keywords:** diabetes mellitus, inflammation, semaglutide, tirzepatide, biomarkers, pentraxin-3, IL-1*β*

## Abstract

Type 2 diabetes mellitus is associated with chronic low-grade inflammation contributing to endothelial dysfunction, metabolic imbalance, and cardiovascular complications. Although semaglutide (SEM) and tirzepatide (TIR) provide important metabolic and cardioprotective benefits, their early anti-inflammatory effects and potential sex-dependent differences remain incompletely understood. This study comparatively evaluated the effects of SEM and TIR on systemic inflammatory biomarkers in a murine model of streptozotocin-induced diabetes mellitus. Thirty BALB/c mice were allocated into six experimental groups according to sex and treatment: control, SEM, and TIR groups (n = 5/group). Diabetes was induced by intraperitoneal streptozotocin administration, followed by treatment with SEM or TIR. Circulating interleukin-1*β* (IL-1*β*) and pentraxin-3 (PTX-3) levels were measured at baseline, one week after streptozotocin administration, and after six weeks of treatment. Control groups exhibited progressive increases in IL-1*β* and PTX-3 levels, indicating sustained inflammatory activation. In contrast, SEM- and TIR-treated animals showed attenuated inflammatory responses characterized by transient or stabilized biomarker profiles. Differential inflammatory responses were observed between treatments and sexes. Male SEM and Male TIR groups demonstrated stable IL-1*β* levels, whereas female treated groups showed persistent elevations, particularly Female TIR animals. PTX-3 responses also displayed differential sex-dependent patterns, with Female SEM animals exhibiting the most stable inflammatory profile. These findings suggest differential early immunomodulatory effects of the two modern antidiabetic drugs, characterized by distinct biomarker responses according to sex and inflammatory marker profile. IL-1*β* and PTX-3 may represent complementary biomarkers for the assessment of early inflammatory activation associated with diabetes mellitus and its cardiometabolic complications.

## 1. Introduction

Diabetes mellitus (DM) is a chronic metabolic disorder arising from either insufficient insulin production or impaired insulin action, leading to persistent hyperglycemia and subsequent complications [1,2,3]. Persistently elevated glucose promotes endothelial impairment via oxidative stress and enhanced formation of advanced glycation end products, reducing nitric oxide bioavailability and favoring collagen cross-linking, low-density lipoprotein (LDL) retention, and atherothrombotic development. In parallel, impaired insulin signaling leads to adipose tissue dysfunction and increased release of inflammatory and prothrombotic mediators, such as tumor necrosis factor-α (TNF-*α*), interleukin-1*β* (IL-1*β*), interleukin-6 (IL-6), and plasminogen activator inhibitor-1, amplifying systemic inflammation and thrombotic susceptibility [4]. Consistent with this, DM is associated with altered circulating inflammatory mediators, including C-reactive protein (CRP), IL-1*β*, IL-6, TNF-*α*, adiponectin, leptin, and other adipocytokines [5].

The International Diabetes Federation reported that 537 million adults were living with DM in 2021, with projections indicating an increase to 783 million by 2045, with substantial socioeconomic impact [6,7]. Cardiovascular disease complications in type 2 diabetes (T2DM) include ischemic heart disease, coronary artery disease, stroke, and peripheral artery disease. These conditions together account for more than half of deaths among affected individuals [8]. Moreover, individuals with T2DM face an approximately two- to four-fold higher risk of developing cardiovascular disease and stroke compared with those without DM, underscoring the substantial cardiometabolic burden associated with this condition [9]. T2DM affects 10.5% of adults aged from 20 to 79 years, which accounts for over 90% of all cases, and is characterized by progressive insulin resistance combined with declining β-cell function. Therefore, T2DM leads to chronic hyperglycemia that induces glucotoxicity, oxidative stress, and mitochondrial dysfunction, further maintaining impairment of β-cell performance [10,11,12,13]. T2DM pathogenesis is multifactorial, involving genetic predisposition, obesity, chronic inflammation, and altered insulin signaling [14].

Management of T2DM involves non-insulin pharmacological agents, including metformin, thiazolidinediones, sulfonylureas, glucagon-like peptide-1 (GLP-1) receptor agonists (GLP-1RAs), dipeptidyl peptidase-4 inhibitors, and sodium–glucose cotransporter-2 inhibitors [13,15]. GLP-1 RAs [such as semaglutide (SEM), liraglutide, dulaglutide, exenatide] are resistant to dipeptidyl peptidase-4 degradation and, by binding to the GLP-1 receptors, promote glucose-dependent insulin secretion, suppress glucagon release, delay gastric emptying, reduce hepatic glucose production, and enhance satiety. These effects collectively improve glycemic control and metabolic regulation, a recognized benefit of long-term treatment [3,13,16,17]. Beyond their glucose-lowering and weight-reducing properties, GLP-1 RAs exert pleiotropic effects that extend to the cardiovascular and cerebrovascular systems. These actions are largely independent of glycemic control and involve modulation of endothelial function, vascular inflammation, oxidative stress, and atherosclerotic processes [18].

SEM is approved for the treatment of T2DM and obesity, with demonstrated cardiovascular benefits [19,20,21,22]. Moreover, SEM ensures cardiovascular protection with a reduction in major adverse cardiovascular events, such as myocardial infarction, cardiovascular mortality, and stroke, through lipid-modulating, anti-inflammatory, and anti-atherosclerotic activity [13,23,24]. In parallel, SEM exerts notable anti-inflammatory actions and supports mitochondrial biogenesis, thereby reducing oxidative stress and contributing to overall metabolic improvement [17].

Tirzepatide (TIR) is a dual glucose-dependent insulinotropic polypeptide (GIP) and GLP-1RA approved for the treatment of T2DM and obesity [25,26,27]. Its pharmacological profile contributes to improvements in blood pressure, triglyceride levels, and LDL cholesterol, supporting its potential to reduce cardiometabolic complications in patients diagnosed with T2DM [14]. TIR represents an innovative incretin-based therapeutic approach with established metabolic and cardiometabolic benefits; however, its gastrointestinal safety profile, particularly in individuals with obesity but without DM, warrants careful evaluation [28,29].

Biomarkers represent objective indicators used to characterize physiological and pathological processes, monitor therapeutic responses, and estimate clinical outcomes. They include measurable molecules in biological fluids; among these, blood-based biomarkers improve diagnosis, prognostic stratification, and therapeutic monitoring, due to their minimally invasive nature and capacity for early disease detection [30,31,32,33].

IL-1*β* represents the most extensively characterized pro-inflammatory mediator within the interleukin-1 (IL-1) cytokine family [34]. It plays a central role in the innate immunity and both acute and chronic inflammatory responses [35,36]. Beyond cerebrovascular disease, IL-1*β* concentrations are significantly elevated in individuals with both type 1 DM and T2DM [34,37]. It is a key mediator of obesity-related inflammation and contributes to hepatic steatosis and pancreatic *β*-cells dysfunction. Even low concentrations exert selective cytotoxic effects on insulin-producing cells [37,38]. Elevated glucose levels induce IL-1*β* production in pancreatic *β*-cells, implicating this cytokine in the pathogenesis of T2DM and cardiovascular risk. Therapeutic inhibition of IL-1*β* reduces the incidence of major cardiovascular events, as demonstrated by clinical evidence from the CANTOS trial [34,39].

Pentraxin-3 (PTX-3) was identified in a broad range of cell types, including vascular smooth muscle cells, myeloid dendritic cells, epithelial cells, and various tumor cells [40,41]. PTX-3 is a long pentraxin and a member of the pentraxin family, which also includes the short pentraxins CRP and serum amyloid P, widely used cardiovascular biomarkers [41]. Structurally, PTX-3 is a 381–amino acid glycoprotein composed of a conserved C-terminal pentraxin domain and a unique N-terminal region that distinguishes it from short pentraxins [40,42]. Unlike CRP, which is synthesized mainly by hepatocytes, PTX-3 is produced locally at sites of inflammation by immune and peripheral tissue cells and increases rapidly within 6–8 h after inflammatory stimulation [40,43]. PTX-3 expression is induced by IL-1, TNF-*α*, nuclear factor kappa B (NF-*κ*B) activation, and is modulated by oxidized LDL, microRNAs, and various pharmacological agents [40,43,44,45]. PTX-3 has emerged as a novel immunoinflammatory biomarker and acute-phase inflammatory molecule, produced locally at sites of inflammation by various cell types, particularly monocytes and macrophages, reflecting endothelial activation and tissue injury [43,44,46,47]. The upregulation has been proposed as an early compensatory response aimed at limiting tissue damage and promoting repair following injury [48].

The present study aimed to comparatively evaluate the effects of SEM and TIR on systemic inflammatory activation in a murine model of streptozotocin (STZ)-induced DM, using IL-1*β* and PTX-3 as circulating biomarkers of metabolic and vascular inflammation. In addition, the study investigated potential sex-dependent differences in the inflammatory response to these novel antidiabetic drugs. By assessing the temporal dynamics of these biomarkers during the early phase of diabetes development, this research sought to explore whether SEM and TIR may exert anti-inflammatory effects, with implications for the prevention of diabetes-associated inflammatory and cardiovascular complications.

## 2. Materials and Methods

The experiments were conducted at the Experimental Research Center for Normal and Pathological Aging, University of Medicine and Pharmacy of Craiova. All national and European regulations regarding the ethical use and handling of laboratory animals were strictly followed. The experimental protocol was approved by the Ethics Committee of the University of Medicine and Pharmacy of Craiova (Approval No. 247/24.06.2025).

### 2.1. Animals

This study included 30 BALB/c mice randomly assigned into six experimental groups (n = 5), as follows:

Group 1: Male Control [Male + STZ + vehicle (VEH)];
Group 2: Female Control (Female + STZ + VEH);
Group 3: Male SEM (Male + STZ + SEM);
Group 4: Female SEM (Female + STZ + SEM);
Group 5: Male TIR (Male + STZ + TIR);
Group 6: Female TIR (Female + STZ + TIR).

The animals were provided by the Animal Facility of the University of Medicine and Pharmacy of Craiova. At the beginning of the study, mice were 7 months old, with an average body weight of 31.16 ± 4.32 g. The animals were housed under controlled environmental conditions, with ad libitum access to food and water, a 12 h light/12 h dark cycle (07:00–19:00), an ambient temperature of 21 °C, and a relative humidity of 60%. Food was withheld prior to STZ administration and before blood sampling for biochemical analyses, while water remained available.

Before each administration, mice were individually weighed using a high-precision electronic scale (CJ-600, HBI Technologies, Phoenix, AZ, USA) to calculate the required dose of the active substance.

One week after STZ administration, mice received TIR (30 nmol/kg) or SEM (200 nmol/kg) administered by subcutaneous injection twice weekly [49] for a six-week period, while control animals received the corresponding VEH in which the active substances were prepared, by an investigator blinded to group allocation. Blinding was maintained throughout all experimental procedures, including blood sample collection and biochemical analyses.

### 2.2. Induction of DM

STZ (140 mg/kg single dose) was injected intraperitoneally within 15 min after preparation in fasted mice [50,51], as STZ is chemically unstable in aqueous solution and undergoes rapid degradation after dissolution, which may reduce its diabetogenic potency and increase experimental variability.

### 2.3. Blood Collection and Serum Preparation

Blood samples were collected from the retro-orbital venous sinus under light anesthesia [isoflurane (Isoflutek, Karizoo, Barcelona, Spain) 2–3% for induction and 1–2% for maintenance in oxygen] at baseline (prior to diabetes induction), 1 week after STZ administration, and after 6 weeks of treatment. The baseline sample (week −1) was collected immediately prior to STZ injection, confirming a normoglycemic state at this time point. The samples were allowed to clot at room temperature and subsequently centrifuged at 3000× *g* for 15 min at 4 °C to obtain serum. The serum was carefully separated and stored at −80 °C until further biochemical analysis.

### 2.4. Reagents

STZ (Catalog No. S0130), citric acid (Catalog No. C1909) and sodium citrate (Catalog No. S4641) analytical grade purity were purchased from Sigma-Aldrich (St. Louis, MO, USA). STZ was freshly prepared immediately before use by dissolution in cold 100 mM citrate buffer (pH 4.5) and protected from light during handling [46,47].

SEM (Catalog No. HY-114118) was purchased from MedChemExpress (Monmouth Junction, NJ, USA), and it was prepared extemporaneously by dissolution in sterile 0.9% sodium chloride solution under aseptic conditions until complete solubilization, in accordance with the manufacturer’s instructions.

TIR (LY3298176) sodium (Catalog No. P1206) was purchased from Selleck Chemicals (Houston, TX, USA), and it was prepared in a vehicle consisting of dimethyl sulfoxide (Catalog No D4540) and corn oil (Catalog No C4648), both purchased from Sigma-Aldrich (St. Louis, MO, USA), in accordance with the manufacturer’s instructions.

### 2.5. Determination of IL-1β and PTX-3 Levels

IL-1*β* levels were determined using a Mouse IL-1*β* ELISA Kit (Catalog No. RAB0274-1KT, Merck, Darmstadt, Germany; sensitivity: 0.005 ng/mL). PTX-3 levels were measured using Mouse PTX-3 Elisa Kit (Catalog No. RAB0857-1KT, Merck, Darmstadt, Germany; sensitivity: 0.6 ng/mL). All assays were performed according to the manufacturer’s instructions.

### 2.6. Statistical Analysis

Statistical analyses were performed using GraphPad Prism (version 9.5.1, GraphPad Software, Boston, MA, USA). Data are presented as mean ± standard deviation (SD). Repeated-measures statistical approaches were employed to evaluate the effects of treatment, sex, and time on inflammatory biomarker concentrations. For IL-1*β*, the presence of missing values precluded the use of conventional repeated-measures analysis of variance (ANOVA); therefore, a mixed-effects repeated measures model was applied. For PTX-3, a two-way repeated-measures ANOVA was performed. In both analyses, treatment group (Control, SEM, and TIR) and sex (male and female) were considered between-subject factors, whereas time was treated as a within-subject factor. The interaction between time and experimental group was evaluated to determine whether biomarker trajectories differed significantly according to treatment and sex. Subject-related variance was estimated within the repeated-measures framework to account for intra-individual variability arising from serial measurements obtained from the same animal over time. When appropriate, multiple comparisons were conducted using Tukey’s post hoc test. Adjusted *p*-values, mean differences, and corresponding 95% confidence intervals (CI) were reported. When sphericity assumptions were not met, the Geisser–Greenhouse correction was applied. Statistical significance was set at *p* < 0.05.

## 3. Results

At week 1 following STZ administration, Male Control animals exhibited mean blood glucose values of 271.4 ± 49.8 mg/dL, Female Control 170.4 ± 8.5 mg/dL, Male SEM 253.9 ± 18.6 mg/dL, Female SEM 170.2 ± 28.4 mg/dL, Male TIR 253.8 ± 45.8 mg/dL, and Female TIR 159.6 ± 8.5 mg/dL, confirming successful hyperglycemia induction across all experimental groups.

### 3.1. IL-1β

#### 3.1.1. Temporal Variations

At baseline (week −1), IL-1*β* concentrations differed significantly between groups, with higher values observed in males compared to females.

**Figure 1:**
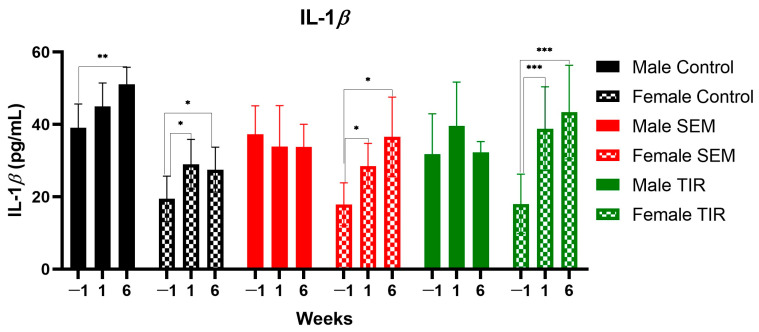
IL-1*β* levels (pg/mL) over time in Male Control, Female Control, Male SEM, Female SEM, Male TIR, and Female TIR groups at weeks −1, 1, and 6. Week −1 represents baseline prior to diabetes induction. Data are presented as mean ± SD. Statistical analysis was performed using Tukey’s multiple comparisons test for simple effects within each group at each time point. Statistical significance was defined as * *p* < 0.05, ** *p* < 0.01, *** *p* < 0.001.

In the Male Control group, IL-1*β* levels showed a progressive increase over time, reaching significantly higher values at week 6 compared to baseline (51.10 ± 4.75 vs. 39.06 ± 6.61 pg/mL; mean difference = −12.04, 95% CI: −18.82 to −5.26; *p* = 0.0020) (Figure 1). No significant differences were observed between week −1 and week 1 or between week 1 and week 6.

In the Female Control group, IL-1*β* levels increased significantly following diabetes induction. Week 1 values were significantly higher than baseline (29.04 ± 6.84 vs. 19.46 ± 6.26 pg/mL; mean difference = −9.58, 95% CI: −17.69 to −1.47; *p* = 0.0228), and this increase remained significant at week 6 compared to baseline (27.52 ± 6.21 vs. 19.46 ± 6.26 pg/mL; mean difference = −8.06, 95% CI: −15.28 to −0.84; *p* = 0.0302) (Figure 1). No significant difference was observed between week 1 and week 6.

In the Male SEM group, IL-1*β* levels remained stable over time, with no statistically significant differences between any time points.

In the Female SEM group, IL-1*β* levels increased significantly from baseline to week 1 (28.54 ± 6.25 vs. 17.94 ± 5.92 pg/mL; mean difference = −10.60, 95% CI: −19.40 to −1.80; *p* = 0.0236) and from baseline to week 6 (36.63 ± 10.96 vs. 17.94 ± 5.92 pg/mL; mean difference = −18.69, 95% CI: −36.10 to −1.27; *p* = 0.0380) (Figure 1). However, the difference between week 1 and week 6 was not statistically significant. 

In the Male TIR group, no significant changes in IL-1*β* levels were observed across time points, indicating a relatively stable inflammatory profile.

In the Female TIR group, IL-1*β* levels showed a marked increase after diabetes induction, with significantly higher values at week 1 (38.84 ± 11.56 vs. 18.04 ± 8.23 pg/mL; mean difference = −20.80, 95% CI: −31.11 to −10.49; *p* = 0.0008) and week 6 (43.44 ± 12.95 vs. 18.04 ± 8.23 pg/mL; mean difference = −25.40, 95% CI: −36.36 to −14.44; *p* = 0.0003) compared to baseline (Figure 1). No significant difference was observed between week 1 and week 6.

Overall, IL-1*β* levels increased over time in the Control groups and in the Female treatment groups, while Male SEM and TIR groups exhibited no significant temporal variation, indicating a sex-dependent inflammatory response.

#### 3.1.2. Between-Group Variations

Between-group comparisons at each time point revealed distinct sex- and treatment-dependent differences in IL-1*β* levels.

**Figure 2:**
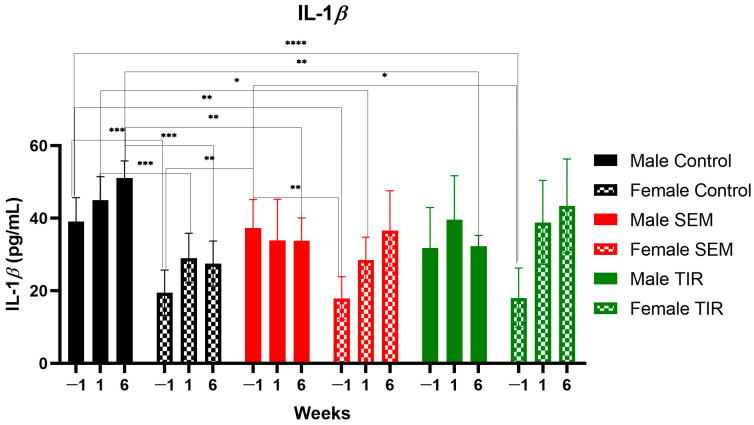
IL-1*β* levels (pg/mL) between Male Control, Female Control, Male SEM, Female SEM, Male TIR, and Female TIR groups at weeks −1, 1, and 6. Week −1 represents baseline prior to diabetes induction. Data are presented as mean ± SD. Statistical analysis was performed using Tukey’s multiple comparisons test for simple effects between groups at each time point. Statistical significance was defined as * *p* < 0.05, ** *p* < 0.01, *** *p* < 0.001, **** *p* < 0.0001.

At baseline (week −1), IL-1*β* levels were significantly higher in male animals compared to females. Male Control animals showed significantly higher values than Female Control (mean difference = 19.60, 95% CI: 9.21 to 29.99; *p* = 0.0009), Female SEM (mean difference = 21.12, 95% CI: 6.71 to 35.52; *p* = 0.0084), and Female TIR (mean difference = 21.02, 95% CI: 13.49 to 28.55; *p* < 0.0001) (Figure 2). Additionally, Male SEM animals had significantly higher IL-1*β* levels than Female SEM (mean difference = 19.40, 95% CI: 7.16 to 31.64; *p* = 0.0057) and Female TIR (mean difference = 19.30, 95% CI: 1.26 to 37.34; *p* = 0.0374) (Figure 2). Female Control animals also presented significantly lower IL-1*β* levels compared to Male SEM (mean difference = −17.88, 95% CI: −29.99 to −5.78; *p* = 0.0081) (Figure 2). No other between-group differences reached statistical significance at baseline.

At week 1, fewer differences were observed. Male Control animals maintained significantly higher IL-1*β* levels compared to Female Control (mean difference = 15.92, 95% CI: 7.64 to 24.20; *p* = 0.0008) and Female SEM (mean difference = 16.42, 95% CI: 4.07 to 28.76; *p* = 0.0136) (Figure 2). No other comparisons between groups were statistically significant at this time point, indicating a partial convergence of IL-1*β* levels following diabetes induction.

At week 6, Male Control animals again exhibited significantly higher IL-1*β* levels compared to Female Control (mean difference = 23.58, 95% CI: 12.90 to 34.26; *p* = 0.0003), Male SEM (mean difference = 17.30, 95% CI: 6.71 to 27.89; *p* = 0.0048), and Male TIR (mean difference = 18.76, 95% CI: 8.13 to 29.39; *p* = 0.0032) (Figure 2). No significant differences were observed between the Male Control and Female SEM or Female TIR groups. Across the remaining comparisons, no statistically significant differences were detected between groups.

Overall, IL-1*β* levels were consistently higher in males compared to females at baseline and week 6, particularly in control animals. Treatment with SEM and TIR appeared to attenuate these differences over time, leading to fewer significant between-group variations at later time points.

The persistent sex-dependent differences observed across time points were further supported by the mixed-effects repeated measures model, which revealed a statistically significant time × sex interaction for IL-1*β* (F(3.354, 21.13) = 4.609; *p* = 0.0105). This interaction indicates that the temporal trajectories of IL-1*β* concentrations evolved differently in male and female animals throughout the experimental period, with males maintaining consistently higher values and a more pronounced progressive increase, while females—particularly in treated groups—showed a delayed but sustained inflammatory response following diabetes induction.

### 3.2. PTX-3

Circulating PTX-3 levels exhibited significant temporal and treatment-dependent variations across experimental groups, reflecting the evolution of systemic inflammation following diabetes induction.

#### 3.2.1. Temporal Variations

**Figure 3:**
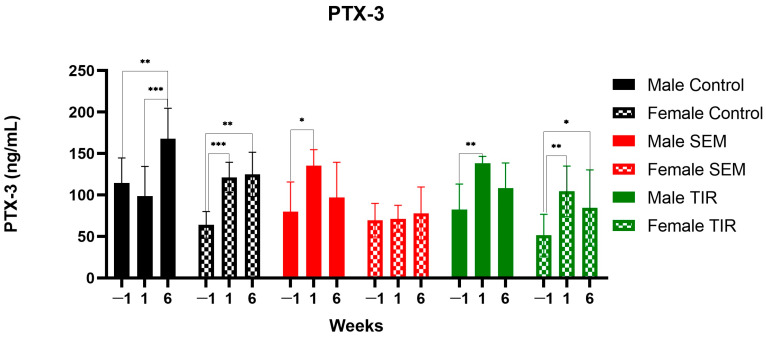
PTX-3 levels (ng/mL) over time in Male Control, Female Control, Male SEM, Female SEM, Male TIR, and Female TIR groups at weeks −1, 1, and 6. Week −1 represents baseline prior to diabetes induction. Data are presented as mean ± SD. Statistical analysis was performed using Tukey’s multiple comparisons test for simple effects within each group at each time point. Statistical significance was defined as * *p* < 0.05, ** *p* < 0.01, *** *p* < 0.001.

In the Male Control group, PTX-3 levels showed a significant increase over time. Although the difference between baseline (week −1) and week 1 was not statistically significant, PTX-3 levels at week 6 were significantly higher than baseline (168.09 ± 36.60 vs. 114.46 ± 30.31 ng/mL; mean difference = −53.63, 95% CI: −88.47 to −18.79; *p* = 0.0066) and week 1 (168.09 ± 36.60 vs. 98.89 ± 35.58 ng/mL; mean difference = −69.20, 95% CI: −92.12 to −46.29; *p* = 0.0001), indicating a progressive and sustained inflammatory response (Figure 3). No significant difference was observed between baseline and week 1.

In the Female Control group, PTX-3 levels increased significantly after diabetes induction. Week 1 values were significantly higher than baseline (121.33 ± 18.24 vs. 64.12 ± 16.05 ng/mL; mean difference = −57.21, 95% CI: −79.70 to −34.73; *p* = 0.0003), and this increase remained significant at week 6 compared with baseline (124.86 ± 26.64 vs. 64.12 ± 16.05 ng/mL; mean difference = −60.74, 95% CI: −93.44 to −28.04; *p* = 0.0023). No significant difference was observed between week 1 and week 6 (Figure 3).

In the Male SEM group, PTX-3 levels increased significantly from baseline to week 1 (135.30 ± 19.47 vs. 79.99 ± 35.84 ng/mL; mean difference = −55.31, 95% CI: −94.60 to −16.03; *p* = 0.0118) (Figure 3). However, no significant differences were observed between baseline and week 6 or between week 1 and week 6, suggesting a transient increase followed by partial normalization.

In the Female SEM group, no statistically significant changes in PTX-3 levels were observed across any time points, indicating a stable inflammatory profile.

In the Male TIR group, PTX-3 levels increased significantly from baseline to week 1 (138.35 ± 8.14 vs. 82.56 ± 30.70 ng/mL; mean difference = −55.79, 95% CI: −84.33 to −27.26; *p* = 0.0017) (Figure 3). However, this increase was not maintained at week 6, as no significant differences were observed between baseline and week 6 or between week 1 and week 6.

In the Female TIR group, PTX-3 levels increased significantly from baseline to week 1 (104.40 ± 30.55 vs. 51.50 ± 25.16 ng/mL; mean difference = −52.89, 95% CI: −89.26 to −16.53; *p* = 0.0089) and remained significantly higher at week 6 compared with baseline (84.38 ± 45.92 vs. 51.50 ± 25.16 ng/mL; mean difference = −32.87, 95% CI: −61.71 to −4.03; *p* = 0.0288) (Figure 3). No significant difference was observed between week 1 and week 6.

Overall, PTX-3 levels increased significantly after diabetes induction in most groups, with sustained elevations in both control groups and Female TIR animals, while SEM- and Male TIR-treated groups showed transient or absent temporal changes, indicating treatment- and sex-dependent modulation of the inflammatory response.

#### 3.2.2. Between-Group Variations

Between-group comparisons revealed several significant differences, particularly at baseline and at week 6.

**Figure 4:**
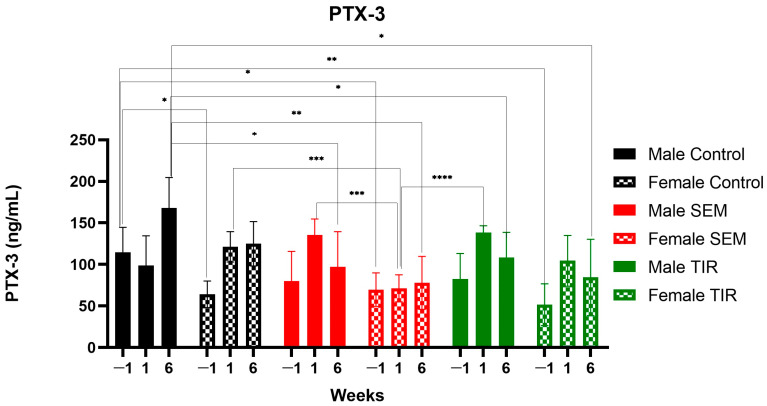
PTX-3 levels (ng/mL) between Male Control, Female Control, Male SEM, Female SEM, Male TIR, and Female TIR groups at weeks −1, 1, and 6. Week −1 represents baseline prior to diabetes induction. Data are presented as mean ± SD. Statistical analysis was performed using Tukey’s multiple comparisons test for simple effects between groups at each time point. Statistical significance was defined as * *p* < 0.05, ** *p* < 0.01, *** *p* < 0.001, **** *p* < 0.0001.

At baseline (week −1), Male Control animals showed significantly higher PTX-3 levels compared with Female Control (114.46 ± 30.31 vs. 64.12 ± 16.05 ng/mL; mean difference = 50.34, 95% CI: 8.73 to 91.96; *p* = 0.0161), Female SEM (114.46 ± 30.31 vs. 69.75 ± 20.25 ng/mL; mean difference = 44.71, 95% CI: 1.54 to 87.88; *p* = 0.0408), and Female TIR animals (114.46 ± 30.31 vs. 51.50 ± 25.16 ng/mL; mean difference = 62.96, 95% CI: 17.05 to 108.90; *p* = 0.0055) (Figure 4). No other between-group differences were statistically significant at baseline. 

At week 1, Female Control animals exhibited significantly higher PTX-3 levels compared with Female SEM animals (121.33 ± 18.24 vs. 71.37 ± 16.19 ng/mL; mean difference = 49.96, 95% CI: 21.62 to 78.30; *p* = 0.0006) (Figure 4). In addition, Male SEM animals showed significantly higher PTX-3 levels than Female SEM animals (135.30 ± 19.47 vs. 71.37 ± 16.19 ng/mL; mean difference = 63.93, 95% CI: 32.51 to 95.35; *p* = 0.0002). Female SEM animals also had significantly lower PTX-3 levels compared with Male TIR animals (71.37 ± 16.19 vs. 138.35 ± 8.14 ng/mL; mean difference = −66.98, 95% CI: −89.09 to −44.88; *p* < 0.0001) (Figure 4). No other comparisons reached statistical significance at this time point.

At week 6, Male Control animals had significantly higher PTX-3 levels compared with Male SEM (168.09 ± 36.60 vs. 96.88 ± 42.68 ng/mL; mean difference = 71.21, 95% CI: 1.71 to 140.70; *p* = 0.0435), Female SEM (168.09 ± 36.60 vs. 77.79 ± 31.84 ng/mL; mean difference = 90.30, 95% CI: 33.89 to 146.70; *p* = 0.0014), Male TIR (168.09 ± 36.60 vs. 108.19 ± 30.44 ng/mL; mean difference = 59.90, 95% CI: 4.44 to 115.40; *p* = 0.0312), and Female TIR animals (168.09 ± 36.60 vs. 84.38 ± 45.92 ng/mL; mean difference = 83.72, 95% CI: 15.14 to 152.30; *p* = 0.0136) (Figure 4). No other between-group differences were statistically significant at week 6.

Overall, between-group differences in PTX-3 were most evident at baseline and at week 6. Baseline differences were primarily driven by higher PTX-3 levels in male control animals compared with several female groups, while at week 6, Male Control animals consistently exhibited higher PTX-3 levels than multiple treated groups. At week 1, significant differences were mainly observed between SEM and TIR groups and their female counterparts, highlighting sex- and treatment-dependent variability in the early inflammatory response.

The considerable inter-individual variability observed within each group, particularly among male controls (SD ≈ 30 ng/mL, ~26% of the mean), aligns directly with the large Subject effect (24.61% of total variance) identified in the two-way ANOVA. This confirms that baseline heterogeneity is not an analytical artifact but a genuine biological feature of PTX-3, and it justifies the use of a repeated-measures design in which each animal serves as its own control across time.

The sex-dependent differences observed across time points were further supported by the two-way repeated measures ANOVA, which revealed a statistically significant time × group interaction for PTX-3 (F(10, 82) = 6.541; *p* < 0.0001), accounting for 17.29% of total variance. This interaction confirms that PTX-3 temporal trajectories evolved differently across experimental groups throughout the observation period, with male animals—particularly Male Control—exhibiting a more pronounced and progressive inflammatory response compared with their female counterparts, while treated female groups, most notably Female SEM, displayed attenuated or absent temporal changes. These findings provide statistical confirmation for the sex- and treatment-dependent modulation of PTX-3 dynamics described above.

## 4. Discussion

In the present research, IL-1*β* levels showed a progressive increase in the Control groups and in the Female treatment groups, while no significant temporal changes were observed in the Male SEM and TIR groups, supporting a sex-dependent inflammatory profile. At baseline and week 6, IL-1*β* concentrations were consistently higher in males compared to females, particularly among Control animals. Notably, SEM and TIR treatment appeared to mitigate these sex-related differences over time, as reflected by the reduced number of significant between-group differences at later time points.

In our study, PTX-3 levels increased significantly following diabetes induction in most groups, with sustained elevations observed in both Control groups and in Female TIR animals. In contrast, SEM- and Male TIR-treated groups exhibited only transient or no significant temporal changes, suggesting both treatment- and sex-dependent modulation of the inflammatory response.

Between-group differences in PTX-3 were most pronounced at baseline and at week 6. At baseline, these differences were largely driven by higher PTX-3 levels in Male Control animals compared to several female groups, whereas at week 6, Male Control animals continued to show consistently higher levels than multiple treated groups. At week 1, significant differences were primarily observed between SEM and TIR groups and their female counterparts, indicating early variability influenced by both sex and treatment.

Several other studies investigated GLP-1 RAs’ effects on inflammatory markers’ levels. The study conducted by Hachuła M. et al. demonstrated a significant reduction in IL-1*β* levels following treatment with GLP-1 RAs, including SEM, suggesting a relevant anti-inflammatory effect on processes involved in atherogenesis [52]. Similarly, Zhang W. et al. demonstrated in their experimental study that SEM significantly reduces IL-1*β* levels in a mouse model of sepsis, this effect being associated with activation of the AMPK pathway and stimulation of autophagy, suggesting a molecular mechanism through which SEM attenuates the inflammatory response [53]. Li et al. have also underlined that SEM led to blockage of reactive oxygen species while improving autophagic mechanisms [54]. Nevertheless, the researchers also proved that SEM ameliorated lipid accumulation and significantly reduced the levels of IL-1*β*, TNF-*α*, and NF-*κ*B, therefore exerting cardioprotective and tissular effects [54]. Also, liraglutide has demonstrated improvement in endothelial protection and tissue recovery after modulating various cytokines and chemokines [55].

In another study conducted by Hachuła M. et al. in human subjects, treatment with dulaglutide was shown to significantly reduce PTX3 levels, therefore influencing local vascular inflammation [44]. Petković-Dabić J. et al. observed in obese patients with T2DM and psoriasis that SEM treatment led to a trend toward reduced IL-1*β* levels (although this change did not reach statistical significance), implicitly suggesting that its anti-inflammatory effects in this context are predominantly mediated through other inflammatory markers, such as IL-6 and CRP [56].

Conversely, Alhowail A. et al. reported in a rat model of T2DM that, although TIR reduced overall neuroinflammation, it did not produce a significant reduction in IL-1*β* levels, suggesting a limited effect on this specific pro-inflammatory cytokine [57].

An important aspect of the present study is that inflammatory biomarkers were evaluated during the early phase following STZ administration, before the establishment of long-standing DM. Although STZ administration induces hyperglycemia within the first days after injection, experimental evidence demonstrates that glycemic severity and metabolic dysfunction continue to evolve progressively over the following weeks, depending on STZ dose, strain, sex, and experimental protocol [58,59,60]. STZ-induced diabetes, therefore, represents a dynamic process characterized by progressive *β*-cell injury, oxidative stress, endothelial dysfunction, and inflammatory activation [50,51]. Consequently, the significant elevations in PTX-3 and IL-1*β* levels observed as early as one week after STZ administration likely reflect an early inflammatory response associated with incipient metabolic dysfunction rather than advanced chronic diabetic pathology. This experimental approach was intentional, as we aimed to investigate whether PTX-3 and IL-1*β* could represent potential early biomarkers of inflammatory activation during the prediabetic or early diabetic phase.

PTX-3 expression is induced by inflammatory cytokines such as IL-1*β* and TNF-*α*, as well as by NF-*κ*B activation and oxidative stress [43,44]. Clinical studies have also demonstrated associations between elevated PTX-3 concentrations and obesity, prediabetes, and newly diagnosed T2DM, supporting its possible role as an early marker of metabolic and endothelial dysfunction [5]. Of particular relevance in this context, Kim et al. [42] demonstrated that PTX-3 deficiency ameliorates STZ-induced pancreatic toxicity through regulation of endoplasmic reticulum stress and *β*-cell apoptosis, and that recombinant PTX-3 directly increases CHOP (C/EBP Homologous Protein, a pro-apoptotic transcription factor activated downstream of endoplasmic reticulum stress) expression and apoptosis in primary mouse islets in a dose-dependent manner. These findings suggest that PTX-3 is not merely a passive marker of inflammation in the STZ model but may actively contribute to pancreatic *β*-cell injury, further supporting the biological relevance of its early elevation observed in the present study.

Similarly, IL-1*β* is recognized as a key upstream mediator of metabolic inflammation and *β*-cell dysfunction [34,36]. Elevated glucose concentrations and metabolic stress stimulate IL-1*β* production through NF-*κ*B activation pathways, promoting *β*-cell apoptosis and impaired insulin secretion [34,38]. Experimental evidence further suggests that inflammatory activation occurs rapidly after STZ administration, before the development of stable chronic hyperglycemia [50,51]. These findings support the concept that inflammatory pathways are activated during the early stages of metabolic dysfunction and may precede overt DM.

In this context, the relatively attenuated or transient inflammatory responses observed in SEM- and TIR-treated groups may suggest an early immunomodulatory effect of incretin-based therapies. This interpretation is supported by recent systematic reviews and meta-analyses demonstrating that GLP-1 RAs significantly reduce circulating inflammatory mediators, including CRP, TNF-*α*, IL-6, and IL-1*β*, beyond their glucose-lowering effects [61]. Additional experimental and clinical studies have reported anti-inflammatory, endothelial-protective, and anti-atherosclerotic effects associated with GLP-1 RAs [18,23]. However, the present study evaluated only circulating inflammatory biomarkers and did not directly assess vascular function, endothelial integrity, or atherosclerotic changes. GLP-1RAs have been shown to attenuate inflammatory signaling through NF-*κ*B modulation [18]. This mechanism may contribute to the attenuated PTX-3 responses observed in the present study, although direct mechanistic investigations were not performed. Furthermore, treatment with GLP-1RAs has been shown to significantly reduce circulating PTX-3 levels in patients with T2DM and confirmed atherosclerosis (*p* < 0.001) [44], a finding directly consistent with the blunted PTX-3 temporal profiles observed in SEM- and TIR-treated animals in the present experimental model.

An important consideration when interpreting the present findings is the distinct pharmacological profile of TIR compared with SEM. Beyond its established metabolic actions, emerging evidence suggests that GIP signaling may exert immunomodulatory effects by influencing macrophage polarization, cytokine production, and adipose tissue inflammation [62]. Consequently, dual GIP/GLP-1 RAs have been proposed to provide additional anti-inflammatory and cardiometabolic benefits beyond those achieved through GLP-1 receptor activation alone [29]; however, the available evidence remains limited [62].

In the present study, TIR attenuated the progression of inflammatory biomarkers compared with control animals but did not consistently demonstrate superiority over SEM. These findings suggest that the potential anti-inflammatory advantages associated with dual GIP/GLP-1 receptor agonism may not uniformly translate across inflammatory pathways, biomarkers, or biological sexes. Therefore, the differential effects of SEM and TIR on inflammatory regulation warrant further investigation.

An important and original observation of the present study relates to the sex-dependent pattern of PTX-3 modulation by incretin-based therapies. Among all experimental groups, the Female SEM group was the only one to exhibit a complete absence of statistically significant temporal changes in PTX-3 across all time points, indicating a stable inflammatory profile throughout the entire observation period. This stands in contrast to the Female Control group, which showed significant PTX-3 elevation already at week 1 (121.33 ± 18.24 vs. 64.12 ± 16.05 ng/mL; *p* = 0.0003), and to the Female TIR group, which demonstrated sustained PTX-3 elevation at week 6 compared with baseline (84.38 ± 45.92 vs. 51.50 ± 25.16 ng/mL; *p* = 0.0288). In male animals, both SEM and TIR produced a transient PTX-3 increase at week 1, followed by normalization at week 6, without significant differences from baseline at the end of the observation period. These findings suggest that SEM may be associated with a more stable PTX-3 profile than TIR in female animals during the study period, a differential effect that may reflect differences in downstream signaling activated by selective GLP-1 RA versus dual GIP and GLP-1 receptor engagement [14,24].

Recent evidence indicates that biological sex can influence responsiveness to GLP-1 RAs, partly through interactions between GLP-1 signaling pathways and estrogens [63]. Furthermore, GLP-1 receptor activation has been shown to suppress NF-*κ*B signaling and reduce the production of multiple inflammatory mediators [64]. Since PTX-3 expression is largely driven by IL-1*β*-, TNF-*α*-, and NF-*κ*B-dependent pathways [65,66], the stable PTX-3 profile observed in Female SEM animals may reflect a more effective suppression of upstream inflammatory signaling. In contrast, TIR acts through dual GIP/GLP-1 RA, and the immunomodulatory effects of GIP signaling remain incompletely characterized and appear highly context-dependent [62]. Although the present study was not powered to directly compare the anti-inflammatory efficacy of SEM and TIR, these observations raise the possibility that sex-specific mechanisms may contribute to the differential PTX-3 responses observed between treatments, a hypothesis that warrants confirmation in larger experimental and clinical studies.

The sex-dependent pattern was even more pronounced for IL-1*β*. In male animals, both SEM and TIR completely prevented the temporal increase in IL-1*β*, with no statistically significant changes across time points in either the Male SEM or Male TIR groups. At week 6, Male Control animals exhibited significantly higher IL-1*β* levels compared to both Male SEM (mean difference = 17.30, 95% CI: 6.71 to 27.89; *p* = 0.0048) and Male TIR (mean difference = 18.76, 95% CI: 8.13 to 29.39; *p* = 0.0032), confirming the anti-inflammatory protective effect of both treatments in male animals. In contrast, female treated groups showed significant IL-1*β* increases from baseline, with the Female TIR group demonstrating the most pronounced and sustained elevations at both week 1 (38.84 ± 11.56 vs. 18.04 ± 8.23 pg/mL; *p* = 0.0008) and week 6 (43.44 ± 12.95 vs. 18.04 ± 8.23 pg/mL; *p* = 0.0003).

The weaker anti-inflammatory responses observed in female animals may be explained by several interrelated biological mechanisms that remain incompletely understood and represent an important direction for future research. Sex hormones, particularly estrogens, are known to modulate immune and inflammatory signaling pathways, including NF-κB activation and downstream cytokine production, which are directly involved in IL-1*β* and PTX-3 regulation [63,67]. Estrogen-mediated modulation of inflammatory signaling may occur through direct crosstalk between estrogen receptors and NF-*κ*B transcriptional activity, a mechanism that can attenuate pro-inflammatory gene expression [68,69].

Pharmacokinetic variability between sexes may further contribute to these differences, as body composition, hepatic metabolism, drug distribution, and clearance can influence systemic and tissue-level exposure to incretin-based therapies [70]. Moreover, sex-specific differences in baseline adipose tissue inflammation, immune cell activation states, and metabolic stress burden may establish distinct inflammatory set-points that modulate treatment responsiveness. Collectively, these biological factors may act in concert to shape the observed sex-dependent inflammatory responses.

The mechanisms underlying these effects remain incompletely defined, particularly with respect to dual incretin receptor agonism. Sex-specific differences in GLP-1 and GIP receptor expression or signaling efficiency may also influence responsiveness to therapies such as SEM and TIR, further contributing to divergent inflammatory outcomes.

A further observation of interest is the dissociation between the PTX-3 and IL-1*β* response profiles across experimental groups. In the Female SEM group, PTX-3 remained completely stable while IL-1*β* showed significant increases, whereas in the Male SEM and Male TIR groups, IL-1*β* was fully stabilized while PTX-3 showed only a transient early elevation. This divergence suggests that PTX-3 and IL-1*β* are at least partially regulated through independent pathways in the context of STZ-induced diabetes and incretin-based treatment. IL-1*β* is primarily produced by innate immune cells and represents a key upstream mediator of systemic metabolic inflammation [34,36], whose modulation by GLP-1RAs is consistent with direct effects on immune cell NF-*κ*B activation [61]. PTX-3, in contrast, is produced locally at sites of vascular and tissue inflammation and may directly participate in STZ-induced pancreatic endoplasmic reticulum stress and *β*-cell apoptosis, adding a tissue-specific regulatory dimension that is distinct from the systemic cytokine cascade [42]. Consequently, PTX-3 may reflect a broader and more persistent pattern of endothelial and tissue activation that is not fully captured by IL-1*β* measurements alone. These findings underscore the value of simultaneously assessing multiple inflammatory biomarkers in experimental models of diabetes-associated inflammation.

The absence of complete normalization of PTX-3 and IL-1*β* levels despite incretin-based treatment should not necessarily be interpreted as treatment inefficacy. STZ induces direct pancreatic cytotoxicity, oxidative stress, and inflammatory injury [50,51], processes that may persist independently of glycemic improvement. Consequently, incretin-based therapies may attenuate the progression or persistence of inflammatory activation rather than fully suppress the acute inflammatory response triggered by STZ administration. Interestingly, control groups generally exhibited sustained inflammatory increases over time, whereas SEM- and TIR-treated animals showed either transient elevations or relatively stable biomarker profiles, supporting a potential modulatory effect on early inflammatory signaling pathways.

## 5. Limitations and Future Directions

Several limitations of the present study should be acknowledged. First, the STZ-induced diabetes model is associated not only with hyperglycemia and *β*-cell dysfunction, but also with direct cytotoxic and pro-inflammatory effects of STZ itself. Therefore, it cannot be fully excluded that part of the observed increases in PTX-3 and IL-1*β* were partially influenced by STZ-mediated tissue injury in addition to the metabolic alterations associated with diabetes development. Additionally, the study did not include a separate non-STZ healthy control group; however, as inflammatory biomarker measurements were performed at baseline (week −1), prior to STZ administration, each animal effectively served as its own prediabetic control within the repeated measures design, allowing post-induction inflammatory changes to be interpreted relative to individual pre-STZ reference values.

Another limitation is represented by the relatively small number of animals included in each experimental group. Although statistically significant differences were detected for several comparisons, the limited sample size increases the potential influence of inter-individual variability and reduces the generalizability of sex-specific and treatment-specific observations. Consequently, the findings should be considered exploratory and require confirmation in larger experimental cohorts.

In addition, the study focused exclusively on circulating inflammatory biomarkers and did not include histopathological, molecular, or immunohistochemical analyses of pancreatic or vascular tissues.

The BALB/c strain exhibits low sensitivity to STZ compared with other commonly used mouse strains, requiring higher doses to induce sustained hyperglycemia [50,51]. Importantly, Wszola et al. [71] demonstrated that in BALB/c mice treated with STZ doses ranging from 140 to 250 mg/kg, fasting C-peptide concentrations remained comparable to control values, while pancreatic insulin-positive areas were reduced by less than 50%. These findings suggest that, in this strain, a 140 mg/kg STZ protocol induces partial *β*-cell dysfunction with preserved residual insulin secretion rather than complete *β*-cell ablation. The present experimental model does not fully reproduce the complex pathophysiology of human T2DM, particularly insulin resistance and obesity-associated metabolic dysfunction. However, it also differs from classical type 1 DM models characterized by near-complete *β*-cell destruction and markedly suppressed C-peptide concentrations [72]. Therefore, the present model may be interpreted as an intermediate form of STZ-induced experimental DM associated with sustained hyperglycemia, residual *β*-cell function, and inflammatory activation, which is appropriate for investigating hyperglycemia-driven inflammatory responses, the primary objective of the current study. Furthermore, the results obtained in this experimental model cannot be directly extrapolated to human T2DM, given the inherent differences between chemically induced diabetes in rodents and the complex chronic metabolic syndrome background of the human disease. Future research employing alternative induction protocols should, however, carefully account for the independent pro-inflammatory effects of dietary fat on circulating biomarkers, particularly PTX-3 and IL-1*β*, which could confound the interpretation of diabetes-specific inflammatory changes.

Several biological factors may underlie the sex-related differences observed in this study. These include hormonal modulation of immune responses, potential differences in incretin receptor signaling efficiency, and sex-dependent pharmacokinetic variability affecting drug exposure, distribution, and clearance. However, these mechanisms remain speculative, as they were not directly investigated in the present work. Therefore, future studies should incorporate mechanistic approaches, including assessment of NF-*κ*B pathway activation, inflammasome signaling (e.g., NOD-like receptor family pyrin domain containing 3), incretin receptor expression, hormonal profiling, and pharmacokinetic analyses, to better elucidate the biological basis of the sex-specific inflammatory responses observed in this model.

## 6. Conclusions

SEM and TIR demonstrated distinct modulatory effects on systemic inflammatory biomarkers in the early phase of STZ-induced DM. Both drugs were associated with attenuated inflammatory biomarker responses, particularly in male animals, where IL-1*β* levels remained stable throughout the experimental period. In contrast, female animals tended to exhibit a less pronounced anti-inflammatory response, especially in the TIR-treated group, suggesting a potential sex-dependent pattern of inflammatory modulation.

PTX-3 and IL-1*β* displayed partially divergent response profiles, suggesting that these biomarkers reflected complementary but distinct inflammatory pathways involved in diabetes-associated metabolic and endothelial dysfunction. The stabilization or transient elevation of PTX-3 observed in treated groups supported the hypothesis that SEM and TIR may modulate inflammatory pathways associated with experimental diabetes. Further studies are required to determine whether these biomarker changes translate into vascular or cardiometabolic benefits.

Larger experimental and clinical studies are required to clarify the molecular mechanisms underlying these sex-specific responses and to determine the translational relevance of PTX-3 and IL-1*β* as early biomarkers of inflammation in DM.

## Data Availability

The original contributions presented in this study are included in the article. Further inquiries can be directed to the corresponding author.

## Abbreviations

ANOVA	analysis of variance
CI	confidence interval
CRP	C-reactive protein
DM	diabetes mellitus
GIP	glucose-dependent insulinotropic polypeptide
GLP-1	glucagon-like peptide-1
GLP-1RAs	glucagon-like peptide-1 receptor agonists
IL-1	interleukin-1
IL-1*β*	interleukin-1 beta
IL-6	interleukin-6
LDL	low-density lipoprotein
NF-*κ*B	nuclear factor kappa B
PTX-3	pentraxin-3
SD	standard deviation
SEM	semaglutide
STZ	streptozotocin
T2DM	type 2 diabetes mellitus
TIR	tirzepatide
TNF-*α*	tumor necrosis factor alpha
VEH	vehicle
